# Gaussian processes retrieval of crop traits in Google Earth Engine based on Sentinel-2 top-of-atmosphere data

**DOI:** 10.1016/j.rse.2022.112958

**Published:** 2022-03-04

**Authors:** José Estévez, Matías Salinero-Delgado, Katja Berger, Luca Pipia, Juan Pablo Rivera-Caicedo, Matthias Wocher, Pablo Reyes-Muñoz, Giulia Tagliabue, Mirco Boschetti, Jochem Verrelst

**Affiliations:** a Image Processing Laboratory (IPL), Universitat de València, C/Catedrático José Beltrán, 2, 46980 Paterna, València, Spain; b Ludwig-Maximilians-Universität München, Munich (LMU), Department of Geography, Luisenstr. 37, 80333 Munich, Germany; c Institut Cartogràfic i Geològic de Catalunya (ICGC), Parc de Montjüic, 08038 Barcelona, Spain; d CONACYT-UAN, 63155 Tepic, Nayarit, Mexico; e Remote Sensing of Environmental Dynamics Laboratory, University of Milano - Bicocca, Milano, Italy; f Institute for Electromagnetic Sensing of the Environment, National Research Council of Italy, via Bassini 15, 20133 Milano, Italy

**Keywords:** Biophysical and biochemical crop traits, Top-of-atmosphere reflectance, Sentinel-2, Gaussian processes (GP), Atmosphere radiative transfer model, Google Earth Engine, Active learning (AL), Hybrid retrieval methods, Euclidean distance-based diversity (EBD), Uncertainty estimates

## Abstract

The unprecedented availability of optical satellite data in cloud-based computing platforms, such as Google Earth Engine (GEE), opens new possibilities to develop crop trait retrieval models from the local to the planetary scale. Hybrid retrieval models are of interest to run in these platforms as they combine the advantages of physically- based radiative transfer models (RTM) with the flexibility of machine learning regression algorithms. Previous research with GEE primarily relied on processing bottom-of-atmosphere (BOA) reflectance data, which requires atmospheric correction. In the present study, we implemented hybrid models directly into GEE for processing Sentinel-2 (S2) Level-1C (L1C) top-of-atmosphere (TOA) reflectance data into crop traits. To achieve this, a training dataset was generated using the leaf-canopy RTM PROSAIL in combination with the atmospheric model 6SV. Gaussian process regression (GPR) retrieval models were then established for eight essential crop traits namely leaf chlorophyll content, leaf water content, leaf dry matter content, fractional vegetation cover, leaf area index (LAI), and upscaled leaf variables (i.e., canopy chlorophyll content, canopy water content and canopy dry matter content). An important pre-requisite for implementation into GEE is that the models are sufficiently light in order to facilitate efficient and fast processing. Successful reduction of the training dataset by 78% was achieved using the active learning technique Euclidean distance-based diversity (EBD). With the EBD-GPR models, highly accurate validation results of LAI and upscaled leaf variables were obtained against in situ field data from the validation study site Munich-North-Isar (MNI), with normalized root mean square errors (NRMSE) from 6% to 13%. Using an independent validation dataset of similar crop types (Italian Grosseto test site), the retrieval models showed moderate to good performances for canopy-level variables, with NRMSE ranging from 14% to 50%, but failed for the leaf-level estimates. Obtained maps over the MNI site were further compared against Sentinel-2 Level 2 Prototype Processor (SL2P) vegetation estimates generated from the ESA Sentinels’ Application Platform (SNAP) Biophysical Processor, proving high consistency of both retrievals (*R*^2^ from 0.80 to 0.94). Finally, thanks to the seamless GEE processing capability, the TOA-based mapping was applied over the entirety of Germany at 20 m spatial resolution including information about prediction uncertainty. The obtained maps provided confidence of the developed EBD-GPR retrieval models for integration in the GEE framework and national scale mapping from S2-L1C imagery. In summary, the proposed retrieval workflow demonstrates the possibility of routine processing of S2 TOA data into crop traits maps at any place on Earth as required for operational agricultural applications.

## 1 Introduction

The quantification of vegetation traits is fundamental to assess the dynamic response of plants to changing environmental conditions (Weiss et al., 2020). Earth observation (EO) sensors in the optical domain enable the spatiotemporally-explicit retrieval of plant traits (Malenovskyý et al., 2019). Among the most auspicious optical EO satellites for the retrieval of vegetation traits currently orbiting the globe is the Copernicus Sentinel family; and in particular the twin constellation of Sentinel-2A and -2B (S2), which are dedicated to terrestrial EO carrying the Multispectral Instrument (MSI). With their relatively short revisit time of up to 2–3 days in mid-latitudes, ground sampling distance (GSD) of 10 m, 20 m and 60 m, and adequate spectral configuration (with 13 bands covering the visible, the near-infrared and the shortwave infrared domains), the S2 constellation can be seen as an ideal data source for agricultural applications at the global scale (Drusch et al., 2012; Malenovský et al., 2012; Vuolo et al., 2018). The S2 sensors together with prior operational satellite missions led to an enormous availability of optical data, thus enabling huge progress towards the development of multiple retrieval methods, e.g., advanced machine learning regression algorithms (MLRAs) and radiative transfer modeling for the estimation of vegetation traits (e.g., Ali et al., 2021; Kganyago et al., 2021; Chrysafis et al., 2020; Darvishzadeh et al., 2019). Yet, all these methods have in common that they rely on a model that converts a measured spectral quantity (usually reflectance) into vegetation traits, which describe status and vitality of vegetation.

Systematic reviews on the taxonomy of retrieval methods are provided in the studies by Verrelst et al. (2015b, 2019a), including discussions on the perspectives of these methods within operational contexts by taking into account both retrieval accuracy and processing speed. Based on a qualitative and quantitative comparison, it was concluded that the so-called hybrid methods provided most confidence to comply with both criteria (Verrelst et al., 2015a,b). Hybrid methods blend the generic properties of physically-based models together with the flexibility and computational efficiency of MLRAs. Within such a scheme, training datasets are generated from radiative transfer models (RTMs) simulations. Then, the MLRA learns the (non-linear) relationship between the pairs of reflectance and vegetation trait of interest. Accordingly, in this hybrid way, a generic model is built that enables processing an image quasi-instantly, thus potentially achieving both accurate and fast retrievals. However, the genericity of the retrieval model depends on the applied RTM and its ability to represent the observed surface types. Artificial neural networks (NN) have long served as the core algorithm in operational processing chains (e.g., Bacour et al., 2006; Baret et al., 2013), yet in the last few decades alternative statistical algorithms emerged, mostly in the fields of decision trees and kernel-based MLRAs (e.g., Houborg and McCabe, 2018; Verrelst et al., 2015b), and more recently also with deep learning algorithms (e.g., Maimaitijiang et al., 2020; Pullanagari et al., 2021). With this trend, retrieval algorithms became more flexible than ever, and excellent performances were reported (e.g., Svendsen et al., 2020).

Despite their diversity, the large majority of developed retrieval methods exploit bottom-of-atmosphere (BOA) reflectance, i.e., after an atmospheric correction algorithm has been applied to acquired top-of-atmosphere (TOA) radiance or reflectance. The rationale for using BOA reflectance is evident: removal of atmospheric effects enables obtaining terrestrial surface spectra. Consequently, variability of the received signal is only driven by biochemical and biophysical properties of the vegetated surfaces, though still some confounding factors (e.g., cloud and atmospheric contamination) may affect the spectra. Although this approach is standard mapping practice, the atmospheric correction step may introduce uncertainty into the final BOA reflectance product (Yang et al., 2020; Laurent et al., 2011b). On the other hand, avoiding atmospheric correction at all would introduce even larger uncertainty. To circumvent both sources of uncertainty, the alternative approach is to upscale training data simulations from canopy to atmosphere levels and derive the vegetation variables directly from TOA reflectance or radiance (Fang and Liang, 2003; Lauvernet et al., 2008; Laurent et al., 2011b, 2011a, 2013, 2014; Mousivand et al., 2015; Shi et al., 2016, 2017; Verrelst et al., 2019b; Bayat et al., 2020; Yang et al., 2021; Estévezet al., 2021). TOA retrieval methods usually rely on the coupling of a vegetation RTM with an atmosphere RTM (Bayat et al., 2020; Mousivand et al., 2015; Verrelst et al., 2019b; Estévez et al., 2021), with the latter explicitly modeling the atmospheric effects on the radiance received by the sensor. The additional parameters introduced by the atmospheric models will likely not lead to an increase of the ill-posed inverse problem as it is well-known from BOA studies employing canopy reflectance modeling (Combal et al., 2003). As demonstrated by a global sensitivity analysis (Verrelst et al., 2019b), atmospheric variables are sensitive in rather different mostly non-overlapping spectral regions, e.g., ozone column concentration or water vapour. Moreover, the spectral signal is affected in a much smaller magnitude by most atmospheric variables compared to the canopy or leaf-level variables, which have a dominant impact in particular in the visible and shortwave infrared regions (Verrelst et al., 2019b). While some studies have demonstrated the coupling with advanced atmospheric RTMs such as MODTRAN (e.g., Bayat et al., 2020; Yang et al., 2020, 2021) for TOA-based retrieval approaches, equally consistent retrieval results can be obtained with simplified atmospheric RTMs such as Second Simulation of a Satellite Signal in the Solar Spectrum (6SV, Vermote et al., 1997) and the assumption of a Lambertian surface (Verrelst et al., 2019b). Estévez et al. (2020) developed a hybrid retrieval workflow by combining leaf-canopy-atmosphere RTMs to retrieve leaf area index (LAI) from BOA and TOA S2 reflectance data. Prototype retrieval models were established by training Gaussian processes regression (GPR, Rasmussen and Williams, 2006) algorithms with simulated data coming from the coupled leaf optical properties model PROSPECT4 (Feret et al., 2008) and canopy reflectance model 4SAIL (Verhoef and Bach, 2007) with 6SV atmosphere RTM (PROSAIL-6SV). In a follow up study, retrieval models for multiple crop traits were established for both S2 BOA and TOA scales leading to the following main findings (Estévezet al., 2021): (1) consistent theoretical performances at BOA and TOA scales, suggesting that hybrid retrieval models can be directly applied to TOA radiance or reflectance data; (2) validation results and associated uncertainties indicated a higher fidelity of the TOA-based retrievals opposed to BOA estimates; (3) canopy variables were more successfully retrieved than leaf variables. Nevertheless, despite the generally applicable hybrid methods, none of the presented GPR retrieval models have yet been introduced into an operational context. Though the development of generic retrieval algorithms is one important aspect of a processing chain, the main challenge is rather to facilitate automated downloading, processing and storage of satellite data and to provide the required hardware.

Hence, when it comes to seamless S2 data processing applicable to any corner in the world, computationally-efficient solutions and processing facilities have to be sought. While GPR processes a single S2 tile reasonably fast (in the order of minutes), processing becomes laborious when time series of S2 tiles must be handled, especially for larger regions. Moreover, preprocessing steps, such as selecting and preparing S2 tiles from the Copernicus data hub (Tona and Bua, 2018), lead to additional run-time. In addition, imposed restrictions to the data hub are a substantial bottleneck, even if processes are fully automated. Altogether, dynamic processing of a vast amount of S2 data requires: (1) migrating towards cloud-computing platforms, and (2) integrating the GPR retrieval algorithms into these platforms. Particularly, in the last few years the Google Earth Engine (GEE) emerged as an attractive high-performance computing platform to enable cloud-based processing of petabytes of S2 data (Gorelick et al., 2017). GEE provides powerful computational capability for planetary-scale data processing and even allows creation and training for well-known machine learning algorithms (Kumar and Mutanga, 2018). However, despite the growing capabilities in advanced machine learning tools in GEE, GPR currently is not part of the GEE environment. Only recently, Pipia et al. (2021) developed a workflow that introduced GPR models into the GEE cloud platform. To make this integration possible it was necessary to review the standard GPR regression formulation to achieve a factorization suitable for a parallel computing, and to implement the corresponding matrix algebra transformation. Secondly, active learning (AL) techniques (Verrelst et al., 2016) were introduced to reduce the original training dataset without loss of information. Though Pipia et al. (2021) demonstrated the feasibility of implementing GPR-based LAI retrieval models into GEE, the workflow was restricted to empirically-trained models that only work with BOA data. The integration of generically-applicable hybrid models of multiple crop traits in GEE with direct application to TOA data, thus without the atmospheric correction step, is still missing.

With this ambition in mind, this study strives for facilitating operational processing of TOA-scale developed GPR retrieval models by enabling implementation into the GEE framework. The following main objectives are identified to make this workflow possible: (1) processing TOA-scale GPR algorithms using AL to provide accurate and lightweight retrieval models for multiple crop traits, (2) implementation of the GPR retrieval models into the GEE environment for smooth crop traits mapping from S2 TOA data over croplands; and finally, with the purpose of moving ahead in exploiting the GEE framework for crop monitoring, (3) to process S2 TOA data into cloud-free crop traits maps at the national scale.

## 2 Materials and methods

**Fig. 1:**
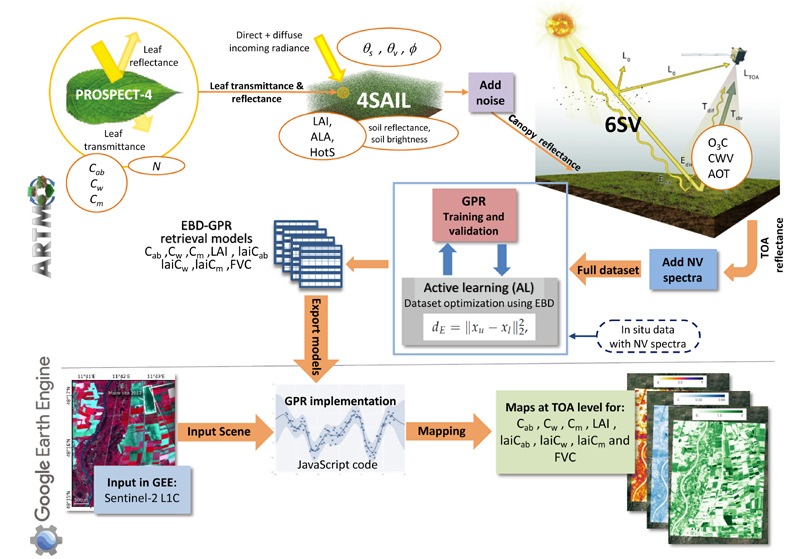
Flowchart of the pursued workflow. Top: Generation of training dataset by coupling of leaf-canopy-atmosphere RTMs for AL optimization and GPR algorithm training. Bottom: Integration of EBD-optimized GPR models (EBD-GPR) in GEE for the retrieval of multiple crop traits from S2 TOA data. See also Table 1 for explanations of model input parameters. Exemplary S2 images used from MNI campaigns (Berger et al., 2020). NV is for non-vegetated.

Our retrieval strategy firstly consisted of the development of hybrid models that estimate crop traits from S2 TOA data within the in-house developed Automated Radiative Transfer Models Operator (ARTMO) software environment (Verrelst et al., 2012c), and then integration of the retrieval models in the GEE platform. The workflow with corresponding models is shown schematically in Fig. 1. The main steps are described in the following sections, being (1) hybrid model development, (2) optimizing the training dataset, and (3) implementation into GEE. Also, additional steps necessary to conduct the methodology are described: ground validation and comparison against SNAP estimates.

### 2.1 Hybrid GPR-based retrieval models

GPR (Rasmussen and Williams, 2006) was chosen as core machine learning algorithm in the hybrid retrieval scheme since it has proven good to excellent performances in numerous studies (e.g., Brede et al., 2020; Mateo-Sanchis et al., 2021; de Sá et al., 2021; Xie et al., 2021; Verrelst et al., 2012a, 2020; Zhou et al., 2018). In the context of recent vegetation traits mapping activities from EO data, these Bayesian non-parametric approaches are to be found among the preferred regression models (Camps-Valls et al., 2018). GPR algorithms are not only straightforward in the training process, they also work well with rather small datasets and adopt very flexible kernel functions for establishing nonlinear relationships between spectral observations and variables of interest (Lázaro-Gredilla et al., 2013). Moreover, final retrieval models provide confidence intervals along with the predictions, which give fidelity of the models’ accuracy as well as insights into robustness of the estimates (Verrelst et al., 2013b). The extensive theoretical description of the GPR algorithms is provided in the original study (Rasmussen and Williams, 2006), and in Camps-Valls et al. (2016, 2018) or Verrelst et al. (2012a, 2015a) in the context of EO data analysis. To enhance understanding of the mathematical background, here we provide a short mathematical description of the input and output GPR formulations. Essentially, GPR technique models the relation between input samples $X\in {\mathbb{R}}^{D}$ (i.e., multispectral S2 pixels) and output observations y∈ℝ (i.e., a specific vegetation trait) as *y* =*f*(**x**) +ϵ, where *ϵ* is an additive Gaussian noise with zero mean and variance $\sigma_{n}^{2}$, and *f*(x) is a Gaussian-distributed random vector with zero-mean and covariance matrix **K**(**x**, **x**), i.e., *f*(**x**) ∼*N* (**0**,**K**). The covariance matrix encodes the similarity between each combination of the input samples **x**_*i*_ and **x***j* using a user-defined kernel function *k*(**x**_*i*_, **x**_*j*_), which takes into account the main statistical properties of the variable to be modeled. For vegetation traits modeling, the asymmetric Square Exponential (SE) is usually preferred due to its capability to (1) successfully approximate smoothly varying functions, and (2) deal with possible asymmetries in the feature space (Camps-Valls et al., 2016). The asymmetric SE defines the covariance kernel function as:

(1) $$k({\text{x}}_{i},{\text{x}}_{j})=\sigma_{s}^{2}exp\left(-\frac{1}{2}{\sum_{b=1}^{D}\left[\frac{{\text{x}}_{i}(b)-{\text{x}}_{j}(b)}{\sigma_{b}}\right]}^{2}\right),$$

where $\sigma_{n}^{2}>0$ represents the output variance while *σ_b_* is related to the spread of the training information along the input dimension *b* in a way that the inverse of *σ_b_* describes the relevance of band *b* in the prediction process: the higher *σ_b_* the lower informative content of *b*. The covariance matrix is completely defined once the kernel’s free parameters and the noise variance, which can be denoted as $\theta =\{\sigma_{n}^{2},\sigma^{2},\sigma_{n}^{2}\}$ with *σ* = [*σ*_1_,.., *σ_D_*], are set. The Bayesian framework of GPR allows estimating the distribution of *f* at any test point x_*_ (i.e., a new S2 pixel) conditioned on the information carried by the training data. According to its formulation, *f*(x_*_) is normally distributed with a mean and variance given by:

(2) $$\begin{array}{l} f(x_{*})=k_{*}^{T}{\left(K+\sigma_{n}^{2}I_{N}\right)}^{-1}y\\ \sigma_{f}^{2}(x_{*})=c_{*}-k_{*}^{T}{\left(k+\sigma_{n}^{2}I_{N}\right)}^{-1}k_{*} \end{array}$$

where *N* is the number of the samples available for the training, k_*_= [*k* (x_*_, *x*_1_),…, *k*(x_*_, *x_N_*)]^*T*^ is an *N* × 1 vector containing the similarity between x_*_ and the training input information, *y* = [*y*_1_,.., *y_N_*]^*T*^ is the training output, and $c_{*}=k(x*,x*)+\sigma_{n}^{2}$. The probability of the observations given the model’s hyperparameters p(y|x,θ) is provided by the marginal likelihood over the function values *f*, whose maximization provides directly the optimum value of *θ* (Rasmussen and Williams, 2006). This optimization procedure is usually referred to as training the GPR (Blum and Riedmiller, 2013). Finally, once optimized *θ* are known, the prediction of *y* for any input x_*_ is given along with its uncertainty by Eq. (2).

For the development of the hybrid models for TOA data we generated a training database with the models PROSPECT-4 (Feret et al., 2008) and 4SAIL (Verhoef and Bach, 2007) (PROSAIL). Subsequently, the atmospheric RTM 6SV was used for upscaling at TOA using atmospheric transfer functions (Vicent et al., 2020). These functions include path radiance, at-surface total solar irradiance due to scattering, total gas transmittance, total upwelling transmittance due to scattering and spherical albedo. The generated functions were then coupled with the PROSAIL simulated vegetation spectra. For these simulations, ARTMO’s TOC2TOA toolbox was employed (v1.03), which was developed to allow coupling surface reflectance with atmospheric simulations to finally obtain TOA radiance data (Verrelst et al., 2019b). The toolbox assumes a Lambertian and homogeneous surface according to the formulation of Guanter et al. (2009). All the parameterization information of the experiment can be consulted in Table 1. The ranges of PROSAIL were set according to the experience of the authors and prior studies (e.g., Verrelst et al., 2021; Berger et al., 2020, 2021; Wocher et al., 2020; Danner et al., 2021). For the 6SV model input parameter ranges and values, also previous studies were consulted (Vicent et al., 2020; Verrelst et al., 2019b), representing generic and globally valid parametrization.

Furthermore, just before coupling with 6SV, white Gaussian noise was injected to the simulated PROSAIL spectral data pool (see Estévezet al., 2021), being common practice for hybrid retrieval workflows in order to introduce more realism to the RTM data and to prevent over-fitting of the models (Brede et al., 2020). Subsequently, 40 spectral samples from non-vegetated surfaces (e.g., water bodies, bare soil or man-made) were added to the training dataset, with a value of zero assigned to all the vegetation variables corresponding to these samples. This step is essential to adapt the models towards processing of full heterogeneous scenes, which are typically characterized by various vegetated and non-vegetated areas. In order to simulate different vegetation states, the key PROSAIL and 6SV input variables were then ranged according to probability density functions obtaining a random dataset of 1'000 simulations of TOA reflectance data. Compared to common sampling sizes ranging from 50'000 to 200'000 samples applied within radiometric look-up table (LUT) inversion strategies (Richter et al., 2009; Omari et al., 2013; Atzberger et al., 2015), this dataset size appears rather small. However, the GPR models applied here cannot handle more than a few thousands of samples due to computational costs scaling cubically with the amount of data. Moreover, as we aimed to provide the models for GEE processing, the training datasets need to be composed of even less than 1'000 samples to guarantee sufficiently light final retrieval models. GPR algorithms tend to be exigent in terms of memory size as all pairs of data need to be stored together with the actual mathematical model (Rasmussen and Williams, 2006; Danner et al., 2021). Therefore, training dataset sizes affects the memory usage of the final models which has to be considered when aiming for storing the models within software toolboxes or for implementation into GEE. This requirement, however, will not decrease the final models’ performance. Recent studies even suggested that 1'000 simulations may be too many for achieving optimal performances in the context of a hybrid workflow (see review in Berger et al. (2021)). This suggests that the representativeness rather than the quantity of a training dataset is the key for optimal GPR-based hybrid models.

### 2.2 Optimization of training data with active learning

When it comes to training data, machine learning regression, such as GPR, works rather passively by receiving labelled data information from which it learns. To obtain successful retrievals, expert knowledge or making use of optimization strategies is required. AL provides a possible optimization by enabling the learner (machine) to collect data according to defined selection criteria (Settles, 2009). Hence, a statistically “optimal way” to select the most meaningful training samples, which reflect the distribution of the measured data, can be performed by the machine itself. Optimization of training samples is well established in classification tasks (see review in Tuia et al., 2011), but less in regression applications (see review in Berger et al., 2021). In the last few years, progressive development of AL resulted in various regression strategies. In this respect, mainly two query frameworks were adapted to solve problems for EO data analysis (Verrelst et al., 2016; Pasolli et al., 2012): (1) *uncertainty* sampling, e.g., He et al. (2014) or Douak et al. (2011), and (2) *diversity* methods, e.g., Lu et al. (2016). Sampling based on *uncertainty* heuristics belongs to the most traditional approaches. Hereby, the algorithm selects those samples out of an unlabeled data pool whose predictions are maximally uncertain (Nguyen et al., 2021). Exemplary methods are variance-based pool of regressors (PAL, Douak et al., 2013) or entropy query by bagging (EQB, Tuia et al., 2011). In contrast, *diversity* methods are based on a dissimilar sampling strategy. A popular technique of this second category is Euclidean distance-based diversity (EBD), which annotates those samples out of the unlabeled data pool that are distant from the already included ones, using squared Euclidean distance (Douak et al., 2013):

(3) $$d_{E}=||x_{u}-x_{l}|{|}_{2}^{2},$$

where *x_u_* is a sample from the candidate set, and *x_l_* is a sample from the training set. All distances between samples are computed and then the farthest are selected. After all, the EBD method removes redundant information from the training database and optimizes it for the retrieval of the requested variable.

From several experimental results and a systematic literature survey, it was concluded that EBD is the most efficient AL method for solving regression problems within EO data analysis (Berger et al., 2021; Upreti et al., 2019; Pipia et al., 2021). EBD not only outperformed other competing AL strategies for the retrieval of multiple crop traits, it was also one of the fastest algorithms in the sample selection process. Hence, the EBD method was chosen for the purpose of this study.

### 2.3 In situ data for active learning and validation

Two field datasets were used for tuning the algorithms and validating the final retrieval models. At first, the Munich-North-Isar (MNI) campaigns in Southern Germany (N 48°16′, E 11 ° 42′) *were* explored and directly involved in the AL procedure as a validation dataset. The long-term consolidated MNI test site is located within communal farmlands owned by the city of Munich. It mainly serves as validation site for the preparation of agricultural algorithms in the framework of the future Environmental Mapping and Analysis Program (EnMAP) (Guanter et al., 2015). The dataset is composed of structural and biochemical crop variables, which were collected concurrently with field spectroscopic measurements on winter wheat (*Triticum aestivum*) and corn (*Zea maize*) during the 2017 and 2018 growing seasons. The two fields were subject to crop rotation, which is common practice in this rainfed agricultural area. Extensive descriptions of the site including visual documentation are provided by Berger et al. (2020); Danner et al. (2019); Wocher et al.(2018). Moreover, the recent study of Estévez et al. (2021) provides the explanation of the same dataset (except leaf dry matter content).

At both fields, a 30 × 30 m area (pseudo EnMAP pixel) was delineated composed of nine elementary sampling units (ESU) of 10 × 10 m. LAI measurements, in [m^2^/m^2^], were performed with the LI-COR Biosciences LAI-2200 device. With the instrument, seven below and one above canopy readings were taken manually and then repeated two times at each ESU. The average of all measurements over the nine ESUs was finally considered as representative. Note that the LAI-2200 instrument is based on gap-fraction and the corresponding software only partially corrects for leaf clumping effects. Hence, the resulting measurements rather correspond to the effective LAI (Jonckheere et al., 2004; Ryu et al., 2010). In addition, the contribution of other plant organs than leaves and non-photosynthetic plant material is included in the measurements. Thus, the term “effective plant area index” would be most appropriate in this context (Leblanc et al., 2005). Nonetheless, the MNI dataset mainly includes green crops and hence, the measured values resemble the (effective) green LAI approximation of the applied RTM, i.e., PROSAIL. For sake of simplicity, we will use the term “LAI” throughout the paper.

Leaf chlorophyll content (*C_ab_*), in [*μ*g/cm^2^], was sampled with a Konica-Minolta SPAD-502 handheld instrument from five leaves per ESU (three points per leaf) considering also different plant heights. The SPAD values were calibrated against destructive measurements of crop leaf chlorophyll content from previous field campaigns at the MNI site. To do so, coefficients of Lichtenthaler (1987) were employed to estimate *C_ab_* from the measurement samples (Danner et al., 2017). Moreover, at each date, two leaves were cut within each ESU (i.e., 18 samples per date). The samples were weighed, packed in bags and brought to the laboratory. There, leaf area was measured using a LI-COR Biosciences LI-3000C scanner attached to the LI-3050C conveyor belt accessory. Final leaf water content (*C_w_*), in [cm] equivalent water thickness, and leaf dry matter content (*C_m_*), in [g/cm^2^], were obtained from the mass difference (per unit leaf size) of sample leaves before and after oven-drying at 105°C (minimum of 24 h) to constant weight. Measured leaf biochemical traits were upscaled to the canopy level by multiplication with LAI, resulting in canopy chlorophyll content, i.e., $\text{LAI}*C_{ab}\;(\text{lai}C_{ab}),$ canopy water content, i.e., $\text{LAI}*C_{w}\;(\text{lai}C_{w})$ and canopy dry matter content, i.e., $\text{LAI}*C_{m}\;(\text{lai}C_{m})$, all given in [g/m^2^]. Table 2 provides an overview of the measured and calculated variables from 2017 and 2018 MNI campaigns, with ranges, mean values and standard deviations.

The second dataset for independent validation of the AL-tuned models was collected during an extensive campaign conducted in central Italy (N 42°49.78′, E 11°4.21′) in the summer of 2018. The site is an agricultural area cultivated with corn and located North of the city of Grosseto. The field sampling was performed within two corn fields characterized by a huge phenological variability related to differences in the sowing dates (i.e., early May and mid of June, respectively) as well as in the irrigation systems. The data were collected during two campaign windows (2–7 July and 31 July-1 August 2018) in correspondence of homogeneous ESUs of 10 × 10 m. LAI was measured in 87 ESUs using either a LAI-2200 plant analyser (LI-COR Biosciences, Lincoln, NE, USA) or digital hemispherical photos collected with a reflex camera (Nikon CoolPix 990, Tokyo, Japan) equipped with a fish-eye lens (Nikon FC-E8 8 mm, Tokyo, Japan), depending on the canopy height and sampling date. During the first campaign, LAI was measured with the hemispherical camera looking downwards within the ESUs with low corn plants (development stage V2-V4) and with the LAI-2200 in the ESUs with high corn plants (development stage V5 or more). In the second campaign all ESUs were measured with the hemispherical camera looking either downwards or upwards depending on the canopy height. The LAI-2200 measurements were performed through four 10 m transects with two-times one above and four below canopy readings. The data were post-processed using the dedicated software and averaged to obtain a representative value for each ESU. The hemispherical photos were collected at the centre and corners of each ESU and the images were processed through the CAN-EYE software (https://www6.paca.inrae.fr/can-eye/) to estimate the average LAI of each ESU. *C_ab_* was measured indirectly within 87 ESUs through SPAD measurements (Konica Minolta, Tokyo, Japan) performed on the last fully expanded leaf (i.e., five readings along each sampled leaf). In addition, *C_ab_* laboratory extractions were performed on the leaf samples collected in correspondence of a subset of 31 ESUs to calibrate the SPAD values. Final *C_ab_* measurements were based on the empirical relationship found between the destructive *C_ab_* measurements and the SPAD readings. *C_w_* and *C_m_* were measured destructively within 31 ESUs. For *C_w_* and *C_m_* quantification, three leaf disks with a 2.2 cm diameter collected from three corn plants per ESU were weighted before and after oven-drying (80°C for 48 h) using an analytical balance with 0.0001 g sensitivity. *C_w_* and *C_m_* were then calculated according to the equations: *C_w_* = (*W_f_*-*W_d_*)/Area; *C_m_* = *W_d_*/Area, where *Wf* and *W_d_* are fresh and dry weights, respectively. Finally, leaf biochemical measurements were upscaled to the canopy-level by multiplication with LAI, alike to the MNI dataset. Hence, for Grosseto, we explored also the four canopy-level variables LAI, lai*C_ab_*, lai*C_w_* and lai*C_m_*.

To obtain the corresponding spectral acquisitions for the in situ data, all available Sentinel-2 Level-1C orthorectified top-of-atmosphere reflectance (L1C) images (maximum 1% cloud cover) within the two growing seasons were requested using the GEE catalog. Ten out of the 13 available Sentinel-2 MSI bands were employed from the images, covering the visible and near-infrared (VNIR) to shortwave infrared (SWIR) domain with central wavelengths of 493 nm, 560 nm, 665 nm, 704 nm, 740 nm, 783 nm, 833 nm, 865 nm, 1610 nm, and 2190 nm. From these scenes, TOA reflectance was extracted at the location of the ESUs, i.e., within the 30 × 30 m pseudo EnMAP pixel.

The above-described simulated training database (Section 2.1) and the in situ dataset from the MNI site were used to tune the retrieval models as follows: firstly, the EBD method was applied to each variable-specific (simulated and measured) dataset, which means one crop trait with corresponding S2 reflectance. To start the EBD procedure, an initial dataset of 5% (i.e., 50 from the 1'000 simulated samples) was randomly selected out of the full data pool (Verrelst et al., 2016, 2020). The procedure was composed of 1'000 iterations, adding each time a new sample to the training dataset. Then, distances between all samples were calculated, keeping only the sample with the largest distance. The new sample was only added to the training data when retrieval accuracy increased as evaluated against the in situ dataset using the root mean square error (RMSE). The process was repeated until all samples of the training dataset were evaluated. In summary, the EBD sampling step was required to identify the optimal model training samples in terms of composition and size. Second, these defined variable-specific EBD-optimized datasets were used for building the EBD-GPR models. In this way, each training dataset had a different sample collection and a different size based on the point of optimal performance of individual variable retrievals. To evaluate the suitability of the optimized EBD-GPR models for implementation into GEE, the usage of full datasets for model building was also tested. For all conducted simulations, the in-house developed ARTMO software framework was used, which includes the MLRA toolbox with an integrated AL module (Verrelst et al., 2020). The 6SV code (6SV2.1) (Kotchenova et al., 2006; Kotchenova and Vermote, 2007) is implemented within the Atmospheric Look-up table Generator (ALG) (Vicent et al., 2020) and coupled to the ARTMO environment (ALG-ARTMO). The full software framework can be freely downloaded at artmotoolbox.com.

### 2.4 Comparison against SNAP retrievals

As an additional evaluation step to assess the suitability of the EBD-GPR models, a comparison exercise was performed using the Sentinel-2 Level 2 Prototype Processor (SL2P) from SNAP (version 7.0) (Weiss and Baret, 2016). The rationale for comparing the results of the hybrid retrieval workflow presented here with these estimates applies to the fact that the SL2P serves as benchmark used by an increasing number of studies and image processing applications (Xie et al., 2019; Pasqualotto et al., 2019; Upreti et al., 2019; Vanino et al., 2018; Danner et al., 2021). Among others, the processor provides LAI, lai*C_ab_*, lai*C_w_* and FVC, thus exclusively canopy-level variables, from Level-2A (L2A) MSI data. SL2P is based on NN algorithms, which are trained over synthetic training datasets generated by the PROSAIL model. Specifically, SL2P employs prior information in the form of truncated Gaussian distributions of input parameters to mimic global conditions (Brown et al., 2021). Detailed information about training dataset sizes and compositions can be found in the algorithm theoretical basis document (Weiss and Baret, 2016). In principle, the workflow is comparable to the one proposed in our study as also a hybrid retrieval strategy was adopted. For the purpose of our study, SL2P NN models were applied for processing BOA (L2A) data into these traits.

For conducting validation, common goodness-of-fit statistics, i.e., root mean square error (RMSE) in variable-specific units, normalized RMSE (NRMSE in %, being RMSE divided by range of observations), and coefficient of determination (R^2^) are provided.

### 2.5 Integration of EBD-GPR models in GEE

The GEE data catalog provides a multi-petabyte collection of widely used satellite imagery, including the complete archives of S2 L1C TOA and L2A orthorectified atmospherically corrected surface reflectance data. On the processing side, the Python Application Programming Interface (API) package *ee* provides functions that allow to extract any available information layer over a specific area of interest (AOI) and process the resulting datasets very efficiently, thus enabling studies at any place on Earth and any time since the launch of Sentinel-2A in 2015. In GEE, for L1C and L2A the datasets are available from 23 June 2015 and 28 March 2017, respectively.

Additionally, the *ee* library provides optimized functions to perform computationally expensive cartographic (mosaicking, compositing, clipping, etc.) and matrix algebraic operations. To circumvent time/space bottlenecks and fully exploit the high-performance parallel computing environment, all these operations must be carried out on server-side of Google processing facilities (Pipia et al., 2021). All in all, a whole study can be bounded to the AOI starting off with the raw images from the available datasets in a few steps. As a demonstration case, two S2 tiles (T32UPU and T32UQU) acquired over the MNI study site on 6 July 2017 were selected. First, the corresponding 10-to-20 m bands were mosaicked and then clipped over the AOI. The maps of the different functional vegetation traits were obtained by importing the corresponding EBD-GPR models generated in ARTMO in the GEE environment and performing the mean value prediction on-the-fly as explained in Pipia et al. (2021). Essentially, the standard formulation of anisotropic-kernel GPR in Camps-Valls et al. (2018) was reorganized in a way to isolate all those terms depending on the model’s hyperparameters and training data, which can be calculated before being imported into GEE. The remaining ones, which account for the mathematical bounds between the new input (i.e., the multispectral S2 imagery to be processed) and specific features of the GPR model, are decomposed into matrix linear algebra operations, which are suited for parallel implementation into GEE. As already mentioned, this computational optimization can be achieved if only functions provided by the *ee* library are used for coding. As a result, the GPR mean value retrievals from a specific S2 tile can be visualized in a few seconds at any zoom level, and the maximum resolution map can be downloaded locally in a few minutes. In Section 3.3, the process is applied to all the S2 TOA EBD-GPR models corresponding to the multiple crop traits over the selected AOI, and in Section 3.4 the resulting maps are compared against the corresponding SNAP estimates. Finally, Section 3.5 shows an example of how to exploit GEE to the fullest, where all the vegetation traits described in Table 2 are mapped at the country scale of Germany. Since mapping the country scale may not fully reveal the details of the obtained trait maps, a few subset maps were additionally generated zooming into specific agricultural regions of Germany at 20 m GSD (see Section 3.5). The GEE codes to run the EBD-GPR models and display the vegetation maps of this study is hosted on the repository https://github.com/esjoal/GEE_GPR_mapping_vegetation.

## 3 Results

### 3.1 Performance of active learning sample selection

**Fig. 2:**
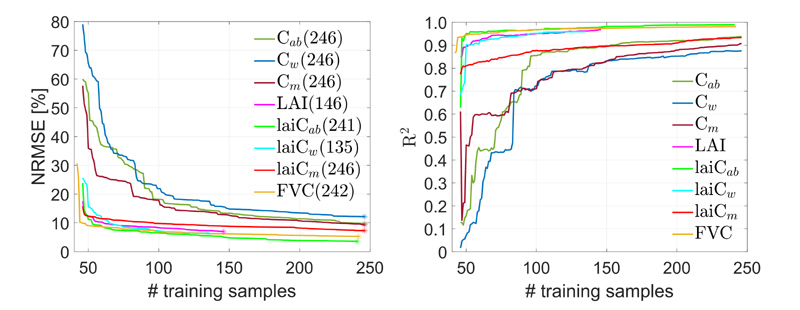
NRMSE (left) and R^2^ (right) for several traits estimations validated against the MNI dataset using the EBD-GPR active learning scheme. The optimal number of training samples for the best performance point (marked with an asterisk) is provided in parentheses. Variable abbreviations can be found in Table 2.

Lightweight GPR retrieval models are essential to avoid memory issues for seamless on-the-fly mapping in GEE. As a workaround, here the AL sampling method EBD was applied to reduce the full training data pool (including 40 non-vegetated spectra) to the most informative samples. The two statistics NRMSE and R^2^ were used to compare the performances across the variables against the MNI field dataset (see Fig. 2). Results improved substantially after adding EBD-selected samples, and then the performances rapidly stabilized, suggesting that newly added samples hardly led to further improvements. The use of RMSE as criterion to keep or discard a sample is reflected in the smoother convergence of NRMSE compared to R^2^. However, in general R^2^ follows the same pattern. All analyzed variables converged to stable accuracy, with particularly high performances for LAI and lai*C_ab_*. Most importantly, Fig. 2 demonstrates that only a few hundred simulations are required to optimize results. For the majority of variables hardly any improvement was achieved in terms of NRMSE after 150 samples. This is especially promising in view of subsequent implementation into GEE given that the lighter the model the smoother the on-the-fly processing. As a result, we obtained training dataset sizes varying from 135 samples (for lai*C_w_*) to 246 (for *C_ab_*, *C_w_*, *C_m_* and lai*C_m_*) (see Fig. 2).

### 3.2 Optimization and validation of retrieval models

**Fig. 3:**
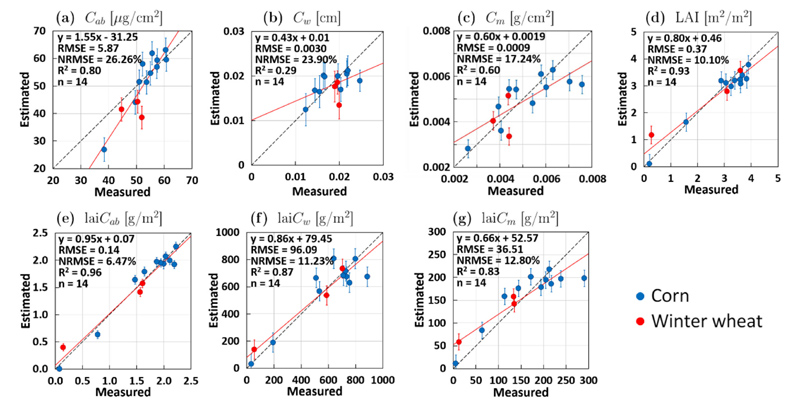
Ground validation for retrieval of biochemical and biophysical crop traits (corn and winter wheat) over the MNI site by the EBD-GPR models from S2-L1C (TOA) reflectance: *C_ab_* (a), *C_w_* (b), *C_m_* (c), LAI (d), lai*C_ab_* (e), lai*C_w_* (f) and lai*C_m_* (g). Measured vs. estimated values along the 1:1-line with associated confidence intervals (1 SD). A trend line (red) was added to represent the pattern of the points.

Based on the results of EBD sample selection, final retrieval models for each variable were established (EBD-GPR models). The exclusion of non-vegetated spectra in the in situ dataset for the ground validation led to slightly poorer validation results (Fig. 3) as opposed to the EBD sample selection (Fig. 2) where non-vegetated spectra were added (see Section 2.3), e.g., NRMSE values for *C_ab_* increased from 10% (EBD sample selection) to 26% (ground validation). This discrepancy can be explained with the fact that the non-vegetated spectra were included in the training dataset for EBD-GPR model building, but excluded for model validation, shown in Fig. 3.

An overview of the different variable retrieval statistics using the full dataset and the EBD-optimized dataset for GPR model building is provided in Table 3. In general, all retrieval results for EBD-optimized datasets improved in comparison to using the full dataset for model building. For instance, in the case of *C_w_* performance increased by 24%, i.e., reducing NRMSE values from 48% (for full dataset) to 24% (for EBD-optimized). For canopy-level variables, improvement was more moderate, as these variables were already well retrieved when implementing the full datasets for model building. Still, the retrieval performance increased, as in case of lai*C_ab_* with highest error reduction from NRMSE = 16% (full) to NRMSE = 6% (EBD). Fig. 3 shows the scatter plots of in situ measured versus estimated variables from the MNI site using the EBD-GPR models. Leaf-level variables are generally moderately validated with, e.g., NRMSE values of 17% for *C_m_* and 26% for *C_ab_*. In contrast, estimated LAI showed high agreement with observed (in situ) data, with NRMSE values of only 10%. The role of LAI also enabled successful leaf-to-canopy upscaling of the leaf variables leading to lower NRMSE values, e.g., 11% and 13% for lai*C_w_* and lai*C_m_*, respectively. Since for FVC no in situ data were available for AL tuning and validation, only the full dataset was applied for model building.

**Fig. 4:**
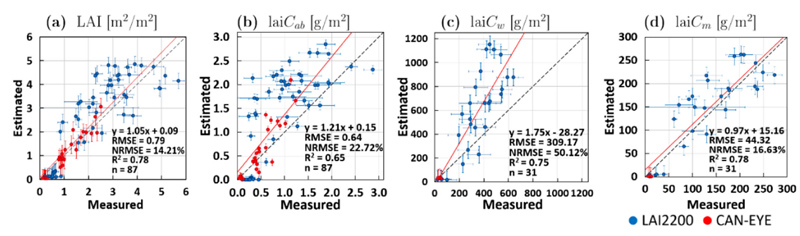
Ground validation of corn for retrieval of canopy-level crop traits over the Grosseto site by the EBD-GPR models from S2-L1C (TOA) reflectance: LAI (a), lai*C_ab_* (b), lai*C_w_* (c) and lai*C_m_* (d). Measured vs. estimated values along the 1:1-line. Horizontal bars indicate SD for ground measurements. Vertical bars indicate associated uncertainty estimates (1 SD) for EBD-GPR model. A trend line (red) was added to represent the pattern of the points. (For interpretation of the references to color in this figure legend, the reader is referred to the web version of this article.)

Fig. 4 provides the independent validation results of the established retrieval models using the corn dataset from the Grosseto site. Scatter plots between measured versus estimated canopy-level traits LAI, lai*C_ab_*, lai*C_w_* and lai*C_m_* are given including goodness-of-fit statistics. Overall retrieval accuracy can be seen as moderate with NRMSE >20% (for lai*C_ab_* and lai*C_w_*) to good with NRMSE <20% (LAI and lai*C_m_*). In particular, lai*C_w_* was significantly overestimated with respect to ground data. This situation can be interpreted as follows by considering both ground data characteristics and retrieval approach. First, the AL-tuning was performed over the MNI dataset, which is characterized by higher *C_w_* values (0.0125–0.025 cm for MNI versus 0.01 to 0.019 cm for Grosseto) that are likely due to the slightly different field sampling protocol. Second, in the MNI dataset the sampling was performed on the entire leaves (i.e., including the leaf veins), while in the Grosseto dataset the leaf disks were only collected from the leaf blades. This may influence the final *C_w_* measured values. Moreover, from author experience, lower values from the Grosseto dataset might also be due to potential loss of water when transporting the samples from field to the laboratory in summer conditions. Finally, it must also be remarked that the over-estimation of LAI affects the results of lai*C_w_* providing consequently higher values with respect to field observation. Compared to fully trained models, the EBD-GPR models even led to slightly lower performance (see Table A.1), for instance in the case of LAI NRMSE = 14.2 (EBD) versus NRMSE = 12.4% (full), or for lai*C_m_* NRMSE = 16.6% (EBD) versus NRMSE = 15.8% (full). Except for lai*C_w_*, these differences in retrieval performance were rather small and the main objective of reducing the models to a feasible size for implementation into GEE was achieved, keeping similar accuracy. The retrieval of leaf-level variables failed (see Fig. A.2) with all traits showing NRMSE >45%. Although, in case of *C_w_* and *C_m_*, we noticed a positive effect of EBD optimization compared to usage of full training datasets for model building. None-theless, our results confirm the difficulty of leaf-level trait estimation from space given a row crop (corn). In row crops, spectral signals transmitted from leaves through the canopy are strongly affected by soil background and structural traits, often leading to rather poor leaf-level retrieval results (Estévez et al., 2021; Xie et al., 2019). The specific heterogeneous canopy architecture of corn fields further complicates the estimation of leaf-level variables, among others, as clumping affects the overall spectral signal (Richter et al., 2010). Considering the independence of the Grosseto dataset, the results provide sufficient confidence of our EBD-GPR models for the estimation of canopy-level variables, though still improvements would be required. Based on these results, we integrated the developed EBD-GPR model into the GEE framework.

### 3.3 Mapping crop traits in GEE

**Fig. 5:**
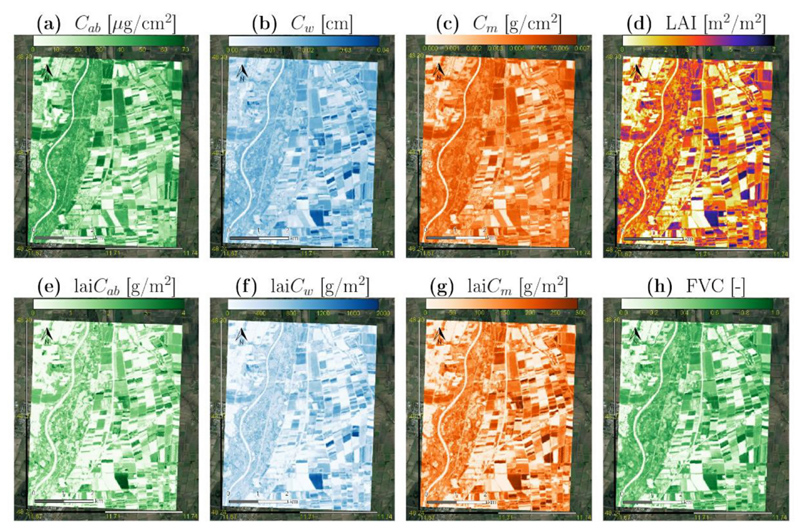
Maps (mean estimates; *μ*) of several crop traits: *C_ab_* (a), *C_w_* (b), *C_m_* (c), LAI (d), lai*C_ab_* (e), lai*C_w_* (f), lai*C_m_* (g) and FVC (h), as generated by EBD-GPR models applied in GEE from S2-L1C data at the MNI test site on 6 July 2017.

Adequate to highly accurate ground validation results of EBD-GPR models proved the feasibility of mapping canopy-level crop traits from S2-L1C data using optimized training datasets for model building and their subsequent implementation into GEE. Transfer of leaf-level trait models may be limited due to failure with the Grosseto data, however, as moderate performances were achieved with the MNI data, these traits will be included for mapping applications. The maps were generated in GEE applying the EBD-GPR models for TOA data products over a subset from the MNI test site on 6 July 2017, as shown in Fig. 5.

**Fig. 6:**
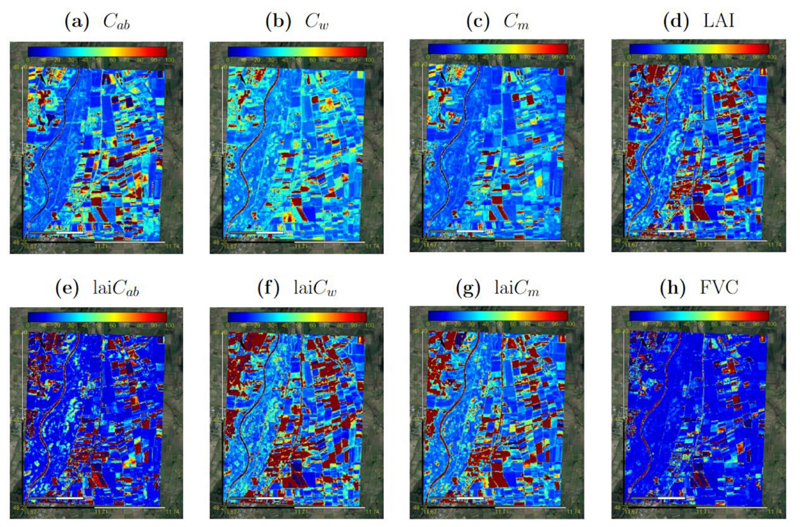
Relative uncertainties expressed as coefficient of variation (CV = SD/*μ* x 100 in %) of several crop traits: *C_ab_* (a), *C_w_* (b), *C_m_* (c), LAI (d), lai*C_ab_* (e), lai*C_w_* (f), lai*C_m_* (g) and FVC (h), as generated by EBD-GPR models applied in GEE from S2-L1C data at the MNI test site on 6 July 2017.

In general, obtained biochemical and biophysical maps are plausible and represent properly the spatial variability of the surface. Both the vegetated and the non-vegetated areas can be correctly identified through their realistic ranges. Even the actual variability of vegetation properties at this point in time is well represented, including fully green and mature crops. The river Isar is also well identified in all maps with close-to-zero values, confirming the overall validity of the retrieval models beyond vegetated surfaces. For comparison, the crop traits maps were also generated with the models trained over the full dataset as shown in appendix section (Fig. A.1). These maps were processed with ARTMO and a visual inspection comparing against maps of optimized models (Fig. 5) does not reveal any substantial difference: the spatial distributions and the dynamic ranges of the traits are similar. In addition, the relative model uncertainty expressed as coefficient of variation (CV = SD/*μ* x 100, in %) was extracted in GEE for all trait maps over the MNI site. Fig. 6 presents the resulting CV maps, indicating high uncertainty for the river and some fields due to unknown spectral signatures, which may be caused by low vegetation cover or particular crop types or reproductive organs not accounted for by the used RTM. For the majority of the fields, however, trustful mapping results were achieved indicating sufficient to high fidelity of the retrieval models.

**Fig. 7:**
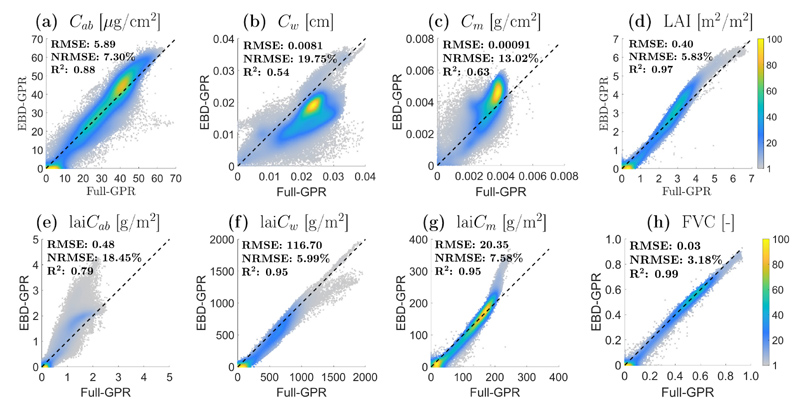
Density scatterplots for comparison of crop traits maps estimated by Full-GPR models (appendix section Fig. A.1) and EBD-GPR models (Fig. 5): C_ab_ (a), C_w_ (b), C_m_ (c), LAI (d), lai*C_ab_* (e), lai*C_w_* (f), lai*C_m_* (g) and FVC (h). Density in %.

For a more exhaustive inspection, scatterplots between EBD-based and those generated using full datasets for the MNI site are shown in Fig. 7. In general, these results show significant moderate to high correlations for all eight variables. Lower correlations are to be found for leaf-level traits, with *C_w_* (*R*^2^ = 0.54) and *C_m_* (*R*^2^ = 0.63), as well as lai*C_ab_* (*R*^2^ = 0.79) (Fig. 7), coinciding with traits of greatest improvement in performance (see NRMSE in Table 3). On the other hand, highest correlations appear for lai*C_w_*/lai*C_m_* (*R*^2^ = 0.95) and LAI (*R*^2^ = 0.97) (Fig. 7), corresponding to the traits with similar performance between the full and optimized models (see NRMSE in Table 3).

### 3.4 Comparison against SNAP SL2P retrievals

**Fig. 8:**
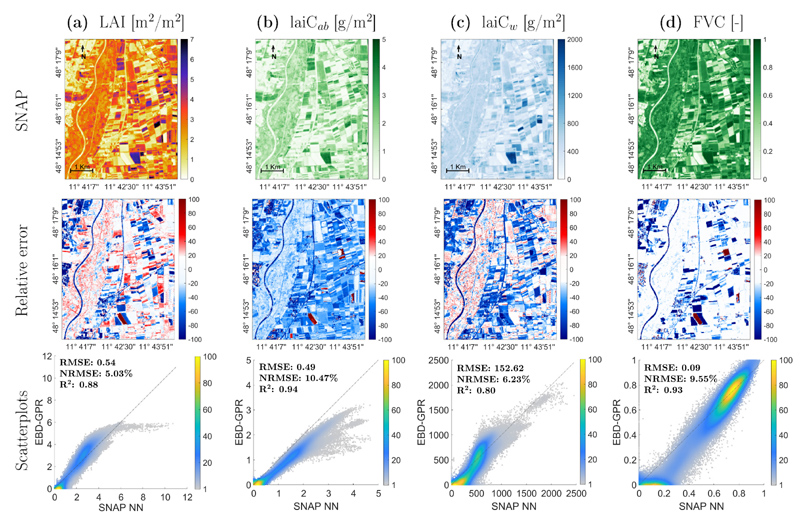
Crop traits maps as obtained by SNAP SL2P NN (Top), relative error maps (Center) and density scatter plots (Bottom) between EBD-GPR (Fig. 5) and SL2P NN, estimated from S2-L2A (BOA) data for LAI (a), lai*C_ab_* (b), lai*C_w_* (c) and FVC (d) over the MNI site on 6 July 2017. Relative errors and density in %.

Apart from the physical validation exercise against the in situ data-sets, the canopy-level models were compared against the same retrievals obtained by the SNAP SL2P models. The maps generated with SL2P NN over the MNI site are demonstrated in Fig. 8 (upper). Overall, the SL2P NN estimate maps look alike to those generated by EBD-GPR in GEE (Fig. 5), obtaining similar spatial patterns. Moderate to good consistency also appeared when comparing the products by means of scatterplots (Fig. 8, bottom): all traits suggest a relatively high correlation between products with an R^2^ between 0.80 and 0.94 for lai*C_ab_*, lai*C_w_*, LAI and FVC. Highest consistency between SL2P NN and EBD-GPR models was achieved for LAI (with lowest NRMSE). For all variables, an over-estimation by SL2P NN can be seen for values close to zero. Strongest discrepancies appear for higher LAI (>6 m^2^/m^2^) and lai*C_ab_* (>3 g/m^2^) estimates.

The relative error maps also reflect these differences between the two products (see Fig. 8, middle). White areas indicate no change within a 20% difference, as it is especcially the case for FVC. Blue colors indicate an underestimation of the EBD-GPR model relative to SL2P NN, clearly observed for lai*C_ab_* with predominant underestimation. In case of LAI, the natural vegetation and some fields showed higher estimates of the GPR-retrieval models opposed to the SL2P LAI maps. The blue color of the river Isar in all maps suggests that SL2P NN does not reach zero values over non-vegetated surfaces as opposed to EBD-GPR model.

### 3.5 Mapping at national scale from TOA data with GEE

**Fig. 9:**
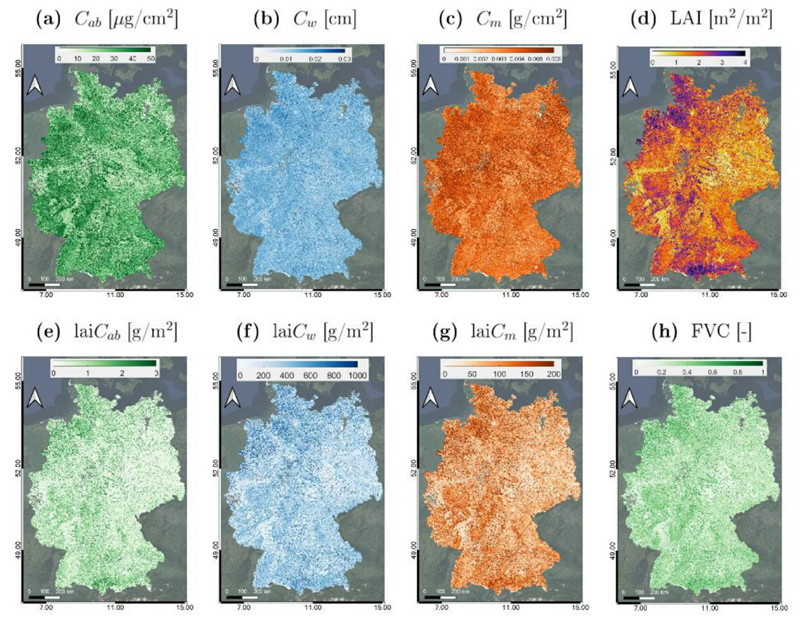
Maps (mean estimates; *μ*) at big scale of several crop traits over the whole of Germany: *C_ab_* (a), *C_w_* (b), *C_m_* (c), LAI (d), lai*C_ab_* (e), lai*C_w_* (f), lai*C_m_* (g) and FVC (h), as generated by EBD-GPR models applied in GEE from S2-L1C data. Time span covers from 01 to 31 July 2017 using median value strategy.

To demonstrate large scale mapping capabilities of GEE, we simulated the crop traits at the national scale for the entirety of Germany. Fig. 9 shows the resulting maps of *C_ab_*, *C_w_*, *C_m_*, LAI, lai*C_ab_*, lai*C_w_*, lai*C_m_* and FVC generated by the developed EBD-GPR models integrated in GEE.

To do this, the procedure described in Section 2.5 was simply applied in a tile-by-tile fashion until the whole country was covered. In this case, we considered a time span instead of a specific date, and finally applied the statistical median estimator to obtain a spatially continuous coverage. Note that this approach allows also to cope with presence of clouds using the quality band ‘Q60’, hence reducing the spatial gaps as much as possible. In general, the time span to be chosen depends on the extension of the area to be imaged as well as its climate properties. For Germany, the acquisitions taken within July 2017 resulted in a collection of 997 scenes spread over 69 distinct tiles. Note that the number of days covered by the input collection is expected to vary with the season and the latitude, but the process can be carried out over any other country worldwide by simply adjusting the input search parameters. The trait maps from each tile were exported as a GeoTIFF file and the national mosaics were finally obtained using GDAL libraries (GDAL/OGR contributors, 2021). Exporting map collections at 20 m from GEE required about 8–9 h and generated an overall data volume of 8 Gb per vegetation trait. Conversely, reducing the spatial resolution to 100 m made the image export task more feasible, both in terms of processing time and file size, dropping off to 10 min and 300 Mb, respectively.

At a glance, the maps show spatial patterns across the country surface and estimation range of variables seems to be correct. However, it must be noted that the maximum boundaries of the color scaling were put lower than of the subset maps. This was necessary because upscaling to 100 m resolution causes aggregation of multiple land covers. Germany, with its characteristic European patchwork landscape, typically consists of multiple small-scale land cover types. This especially holds true in July where cereal fields normally reach senescence. As the crop traits retrieval models were trained for green vegetation, upscaling to a coarser scale leads to aggregation, achieving systematically lower values for all crop traits.

**Fig. 10:**
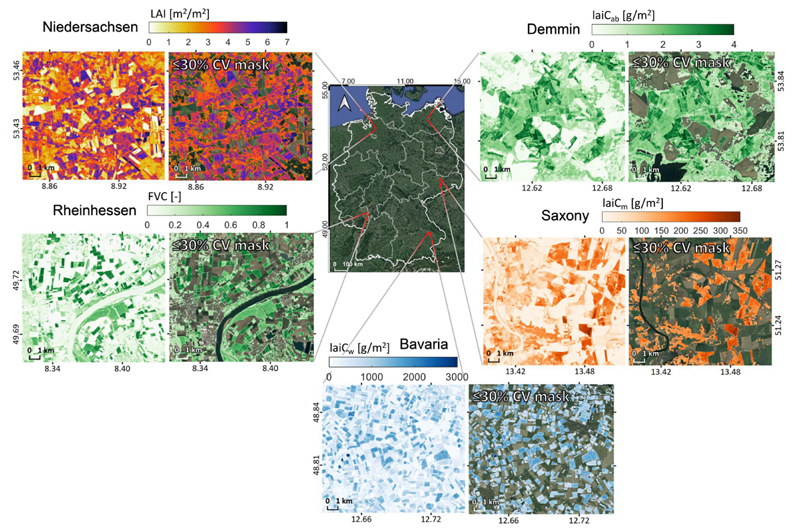
Subset maps for five selected German regions with typical agricultural usage in 20 m spatial resolution (left maps). Uncertainty provided by the EBD-GPR models was used to mask out areas with more than 30% of relative uncertainty (CV) (right maps).

For a better interpretation of estimated traits, subset maps of 20 m spatial resolution are provided for five selected German regions characterized by specific agricultural conditions and with a relatively high share of pastures and croplands (see Fig. 10). For each region, one canopy-level trait was selected as exemplary case study on 6 July 2017. Ground reference data were not available for these regions, but instead the same maps are again shown only for the retrievals that fall within a threshold of 30% relative uncertainty. These mapping serves only for demonstration and interpretation is solely be done by plausibility and given associated uncertainty estimates. Mapping of LAI was conducted for an area in Niedersachsen (Lower Saxony), being one of the most important locations for agriculture in Europe (Rega et al., 2020). The federal state is characterized by intense agricultural usage with cereals, extensive grasslands, sugar beets and potato crops, as is reflected in the relatively high LAI values compared to the rest of Germany (see also Fig. 9). Canopy chlorophyll content was calculated for an area around Demmin in Mecklenburg-Vorpommern, which was identified as a remote sensing test site for validation and calibration (Gerighausen et al., 2007). In this region, mainly cereals are cultivated in the large fields, as indicated by the almost senescent coverage. Still, several fully green fields can be easily distinguished, which may correspond to corn or sugar beet, being most common crops. Mapping of FVC is provided for a small area in Rheinhessen (Rheinland Pfalz), being the largest German wine-growing region with around 26'300 ha of vineyards. Here it can be noted that the specific canopy architecture of the vineyards rather leads to lower FVC values. Canopy dry matter content was simulated for an agricultural area close to the river Elbe in Saxony. In this example, the within-field patterns of the crops reveal some irregularities, probably caused by sandy soil streams (postglacial sandy deposits) due to previous meanders in the floodplain. Lastly, canopy water content estimations are demonstrated for the region around Irlbach, Bavaria. Also here, cereal crops typically are about to reach mature (senescent) growth stages at this time of the year and thus some fields already exhibit low values. Additionally, also the associated uncertainties provide relevant information. When comparing full traits maps against the masked maps, it can be observed that for all traits medium to high estimates are consistently preserved while low retrievals are masked out. The latter can be explained by the combination of a low estimate with some absolute uncertainty (SD), leading rapidly to a relative uncertainty above 30% (CV = SD/*μ* x 100, in %).

## 4 Discussion

With the ambition to automate the mapping of a variety of crop traits from satellite data, an optimized hybrid retrieval processing strategy was developed that can be implemented into the GEE framework. The core idea of this strategy is that the retrieval models can be directly applied to S2 TOA reflectance data without the need of preprocessing steps, such as downloading images or atmospheric correction. In the following, performances of AL optimization (Section 4.1), towards operational TOA-based retrieval (Section 4.2), and finally encountered challenges and future opportunities of the GEE workflow (Section 4.3) are discussed.

### 4.1 Performance of active learning optimization

A key finding of the workflow presented here is the substantial improvement achieved by the implemented AL strategy. Using the EBD sampling method for optimal reduction of the training databases led to moderate (leaf-level traits) to high (canopy-level traits) performances with relatively few simulations applied on the MNI data (see Table 3), confirming earlier experiences with AL (Verrelst et al., 2016, 2020; Berger et al., 2021; Pipia et al., 2021). The increase in retrieval accuracy can be explained by the positive effects of this intelligent sampling method, which decreases redundancy but keeps spectral variability of reflectance datasets. Since the AL sampling selection is run against in situ data, it is also essential that the field dataset covers a sufficiently broad range of vegetation states. Hence, the collection of good quality field data remains an important part of the retrieval algorithm development anticipating the need of extended validation datasets covering multiple crop types and growth stages. In this respect, the transfer of the established EBD-GPR models only achieved a limited accuracy for the Grosseto field dataset, since the application of leaf-level models failed to provide trustful estimates. Besides this, when it comes to processing full scenes, typically characterized by multiple land covers, the retrieval models need to be adapted to diverse spectral surfaces. This can be easily achieved by adding non-vegetated spectra (i.e., bare soil, water, man-made surfaces, etc.) to the training dataset. Training the models some-what less specialized towards exclusively vegetation surfaces has the drawback that slightly poorer validation results are obtained against in situ data when these additional spectra are part of the learning process (around 10% NRMSE in our study). Nonetheless, broadening the training dataset to non-vegetated spectra is an essential part of developing generally applicable hybrid retrieval models for processing heterogeneous landscapes into vegetation functional traits (De Grave et al., 2020). After all, generic models are strived for, i.e., ensuring valid estimates over full satellite scenes.

Model size reduction is an essential step for implementation into GEE. Despite that the training datasets were heavily reduced as opposed to the full dataset (75% or more), retrieval accuracies were superior for all variables from the MNI site (see Table 3) and on the same order for the canopy variables from the Grosseto site (see Table A.1) when using EBD-optimized models, though a relatively small field dataset was employed for AL tuning. These results suggest that GPR models benefit rather from representative training samples than from quantity, as was also noted in Berger et al. (2021). Therefore, it is expected that future hybrid retrieval models will converge towards an optimization of both representativeness and quantity of training datasets, as it can be solely achieved by means of AL.

Apart from validation against local field datasets, the canopy-level retrieval models were also compared to the same estimates as obtained by the models embedded in the SNAP Biophysical Processor toolbox over the MNI test site. The SL2P NN retrievals can be considered as reference used by diverse studies to evaluate if their own products meet user requirements over agricultural environments or other eco-systems (Amin et al., 2021; Brown et al., 2021; Mourad et al., 2020). Here we obtained overall similar maps from SL2P compared to the EBD-GPR retrieval models, as confirmed by scatterplots (Fig. 8). Nevertheless, processing a single image is likely insufficient to draw conclusions about the quality of these products. Such comparison study could be extended to multiple sites and repeated over several dates to confirm this consistency over space and time in a future study. As demonstrated in this work, with GEE in principle these products can be easily obtained anywhere in the world, and at a large scale. The key message of this comparison exercise is that TOA-based retrieval models can be straightforwardly developed showing high consistency with the estimates provided by the SNAP SL2P toolbox.

### 4.2 Towards operational TOA-based retrieval

The S2 L1C TOA reflectance products, being acquired since July 2015, satisfy specifications for radiometric performance with adequate radiometric calibration uncertainty below 3% and uncertainty around 5% in the worst case (Djamai and Fernandes, 2018). A key advantage of working with L1C data is its longer historical availability than L2A data. For instance, in GEE or SentinelHub, L1C data is available since June 2015, while for L2A that is only the case since March 2017, which corresponds to the date the official product started to being distributed by ESA using the Sen2Cor processor. Yet, in other official data hubs, such as the USGS portal, only S2-L1C data is offered. Having efficient models applicable directly to L1C imagery easily allows to create and process longer, consistent and thus more meaningful time series of multiple traits.

Apart from a few studies exploring TOA-based LAI retrieval (Kganyago et al., 2020; Estévez et al., 2020), we are not aware of other works exploring the S2 TOA product for the estimation of (multiple) crop traits. An explanation why this approach has been left aside may be that S2 offers a sound atmospherically-corrected reflectance product (L2A) that has proven to be suitable for the retrieval of a range of vegetation traits (Upreti et al., 2019; Vanino et al., 2018; Novelli et al., 2019; Vuolo et al., 2016). Nevertheless, atmospheric correction is a critical task and discrepancies have been reported depending on the applied atmospheric correction method, spectral bands and land cover type (Sola et al., 2018; Vanino et al., 2018; Doxani et al., 2018), which could be avoided when directly processing TOA data into vegetation properties. Note that the TOA retrieval approach presented here was performed with ARTMO’s TOC2TOA toolbox assuming a Lambertian surface. Though this approximation may introduce small additional uncertainty (Wang et al., 2010), it was shown to provide accurate retrievals for flat surfaces (Verrelst et al., 2019b; Estévez et al., 2020, 2021) and has been also adopted by other studies (e.g., Gómez-Dans et al., 2016). Hence, TOA-based retrieval algorithms represent an appealing alternative, e.g., (1) for experimental missions where BOA reflectance is not provided as standard product, (2) for new-generation imaging spectrometer missions where due to hundreds of contiguous spectral bands, atmospheric correction becomes highly challenging over the full spectral region (Vibhute et al., 2015), (3) for airborne or drone missions where new atmospheric correction procedures have only recently been proposed (e. g., Schläpfer et al., 2020), which despite the promising results need further validations.

Though we demonstrated the processing of S2-L1C data, it must be emphasized that with the hybrid modeling concept, training data can be simulated for any optical sensor data from the visible to the shortwave infrared using the ALG-ARTMO software framework. This implies that the retrieval models can be built to process whatever type of TOA reflectance or radiance into crop traits, as long as the precondition of clear sky during the sensor overpass is given. Future studies could fruitfully explore this approach further by developing and validating TOA-based retrieval models for different crop types, locations and sensors.

### 4.3 Challenges and opportunities with GEE

With the advent of cloud-computing platforms, such as GEE, satellite-based vegetation or crop trait mapping progressed towards a new paradigm (Wagemann et al., 2021). Thereupon, we move away from desktop-based processing to cloud-based image analysis. The paradigm shift is triggered, among others, through the enormous increase of high temporal resolution (S2) data available in GEE, allowing the automatic processing of precise large-scale maps of agricultural fields. In GEE, entire data collections of multiple EO missions from medium-to-coarse spatial resolution are available online for free, and the user-friendly JavaScript/Python libraries (e.g., Wu, 2020) allow launching computational-demanding processes over distributed computing platforms. Besides, efficient mosaicking tools enable to deal with raster and vector information at once for selecting specific areas to be studied, or extend the analysis to nation-wide coverage (Li et al., 2019; Tamiminia et al., 2020). The diversity of terrestrial mapping applications is rapidly expanding, with the large majority of GEE-based studies in the domains of land cover classification (e.g., see review Amani et al., 2020), but also vegetation properties mapping applications are reported. These quantification studies either rely on vegetation indices (e.g., Thieme et al., 2020; Zhen et al., 2021) or on data-driven MLRAs such as random forest models (e.g., Campos-Taberner et al., 2018; Kang et al., 2021). Progressing along this line, only recently the implementation of GPR models into GEE was introduced by Pipia et al. (2021). Despite the fact that GPR is a highly competitive MLRA and has appealing advantages over others with the provision of associated uncertainty estimates (Verrelst et al., 2012a,b, 2013a,b, 2015a), its integration in GEE is not straightforward. Instead, the GEE contiguous memory allocation restrictions associated with the usage of the Matrix algebra operations involved in GPR estimation required workarounds (Pipia et al., 2021). First, the size of the GPR training data had to be reduced using AL. Second, GPR formulation terms independent of the input multispectral imagery had to be precalculated to avoid redundant operations, and third, all pixel-dependent terms were expressed in parallelizable operations. In our study, we followed these main steps for the implementation of the multiple trait models, here directly from TOA reflectance data. Unfortunately, it is impossible to quantify resources made available for the processing in GEE. However, according to our empirical experience, resources seem to be related to the number of users using GEE simultaneously. When it comes to implementing GPR formulation into GEE, we heavily back up on matrix algebra operations to carry out calculations, such as multiplication, inversion, and transposition over spectral bands managed as matrices. To do so, GEE obliges the user to convert (casting) imagecollection-type data to array data-type, which imply using physically contiguous memory for their storage.

Our example of nation-wide crop traits estimations over Germany exemplifies the potential of GEE-based mapping, including the GPR capability of providing uncertainties (see Fig. 10). The latter information can be used to mask out those fields or areas whose estimates provide highest ambiguity probably due to unknown spectral signatures not present in the training database. Therefore, uncertainty information extracted from GEE gives our workflow a new perspective, as it can be used as a quality information for follow-up analysis and applications. Since the detail of 20 m resolution was not visible when printing out the maps, it was decided to process at 100 m resolution. The coarsening step also simplified the processing, in terms of memory handling and processing speed. However, coarsening implies aggregation of patchwork landscape, leading to a trend of lower maximal values than at the 20 m resolution. The impact of heterogeneous landscapes on scaling is well understood, e.g., upscaling mechanisms have been more thoroughly studied by Chen (1999);Garrigues et al. (2006); De Grave et al. (2021); Meier et al. (2020). However, a deeper analysis goes beyond the scope of the present study. In principle, crop properties can be quantified anywhere, over large areas and over multiple years at any scale with this cloud-computing framework. By providing such information for crop monitoring, our proposed method could assist in recent progress towards the Agriculture 4.0 era (Arauújo et al., 2021).

Nevertheless, this approach requires some practice on dealing with memory issues. As a guideline, we recommend: (1) to keep the GPR models as small as possible whilst maintaining adequate performance, (2) to coarsen the S2 data for larger-scale mapping, (3) when filtering collections of images, first apply more selective filters, (4) if memory problems arise, export the results for later processing, and (5) use the most computational expensive GEE algorithms and operations with precaution (e.g., clipping). Although there is a “Profiler” feature in the GEE JavaScript code editor, which displays information about the resources (CPU time and memory) consumed by specific algorithms and other parts of a computation, it is difficult to determine the maximum workload to avoid the aforementioned memory errors as there is no specification on the characteristics of the cluster of computing nodes (e. g., number of working nodes available per user or hardware specification). This feature enables the detection of the most demanding operations performed in the scripts in terms of optimization and debugging.

Given this all, we are only at the onset of cloud-based satellite data processing. In the coming years, crop traits retrieval will be further facilitated within the free-to-use GEE computational infrastructure, enabling seamless mapping over larger areas and multiple time periods (Tamiminia et al., 2020). With the implementation of EBD-GPR models into GEE, in principle any kind of variable can be routinely retrieved. This implies that apart from the crop traits presented here, not only other vegetation models (e.g., related to non-photosynthetic vegetation, Amin et al., 2021), but also those targeting other land cover types, such as models dedicated to the quantification of water variables (e.g., Ruescas et al., 2018) or soil properties (e.g., Vaudour et al., 2019) can be provided. Furthermore, the presented workflow can serve as a foundation for the computation of higher-level products, e.g., time series processing for the calculation of phenology metrics (e.g., Misra et al., 2020; Htitiou et al., 2020; Salinero-Delgado et al., 2021), fusion or assimilation of multiple products (e.g., Pipia et al., 2019; Schreier et al., 2021; Sadeh et al., 2021). At the same time, although this work focused on the processing of S2 TOA data, it must be emphasized that essentially the EBD-GPR retrieval models can be developed for any optical sensor data with the ALG-ARTMO software framework. A similar vegetation properties retrieval approach based on Sentinel-3 TOA data is already in preparation, and models for other optical data sources (e.g., Landsat or MODIS), if available in GEE, can likewise be prepared.

## 5 Conclusions

This study presents an innovative workflow for operational mapping of multiple crop traits from top-of-atmosphere S2 data. We optimized a hybrid retrieval method by implementing AL sampling to establish lightweight GPR retrieval models for processing into the cloud-computing GEE framework. The workflow included the following essential steps: (1) generation of training datasets by coupled leaf-canopy-atmosphere RTMs in the ARTMO software environment, (2) applying EBD-based sampling for building small but efficient training datasets and tuning the models towards real spectra, (3) adding non-vegetated spectra to the training database to ensure overall applicability of final models to process full heterogeneous satellites scenes, and finally (4) integration in GEE to obtain trustful regional and nation-wide maps of multiple crop traits.

Substantially higher accuracy was obtained with the EBD-optimized GPR models for the estimation of all traits over the MNI site including overall lower uncertainties than when fully sampled datasets were used for model building. EBD-optimized models further allowed to map canopy-level traits from an independent test site, while mainly preserving estimation accuracy. Moreover, plausible and consistent maps were obtained in comparison to those provided by the SNAP SL2P toolbox, suggesting high transferability of the proposed hybrid method. Having the EBD-GPR models customized for GEE integration, possibilities are opened to routinely map these traits anywhere in the world. Though nation-wide maps were processed at 100 m spatial resolution, also more detailed maps of 20 m were generated for dedicated sites. In addition, relative uncertainty provided by the EBD-GPR models was used for masking out most uncertain areas providing spatially detailed information about the fidelity of the retrieval models. Altogether, we can conclude that the workflow described here presents a promising path towards operational mapping of essential crop traits with the high-performance computing capacity of GEE, to be used for a multitude of agricultural applications supporting management decisions from farm to regional levels.

## Supplementary Material

Appendix A

## Figures and Tables

**Table 1:** Parameterization of leaf (PROSPECT-4), canopy (4SAIL) and atmosphere (6SV) models, with notations, units, ranges and distributions of inputs used to establish TOA synthetic reflectance databases. $\bar{x}$: mean, SD: standard deviation. LHS: Latin hypercube sampling.

Model variables		Units	Range	Distribution
*Leaf variables*: PROSPECT-4				
*N*	Leaf structure parameter	unitless	1.3–2.5	Uniform
*C_ab_*	Leaf chlorophyll content	[*μ*g/cm^2^]	5–75	Gaussian ($\bar{x}$: 35, SD: 30)
*C_m_*	Leaf dry matter content	[g/cm^2^]	0.001–0.03	Gaussian ($\bar{x}$: 0.005, SD: 0.001)
*C_w_*	Leaf water content	[cm]	0.002–0.05	Gaussian ($\bar{x}$: 0.02, SD: 0.01)
*Canopy variables*: 4SAIL				
LAI	Leaf area index	[m^2^/m^2^]	0.1–7	Gaussian ($\bar{x}$: 3, SD: 2)
*α_soil_*	Soil scaling factor (brightness)	unitless	0–1	Uniform
ALA	Average leaf angle	[°]	40–70	Uniform
HotS	Hot spot parameter	[m/m]	0.01	–
skyl	Diffuse incoming solar radiation	[fraction]	0.05	–
FVC	Fractional vegetation cover	[fraction]	0.05–1	–
*Atmospheric variables*: 6SV				
*O*3*C*	O_3_ Column concentration	[amt-cm]	0.25–0.35	LHS
*CWV*	Columnar Water Vapour	[*g*. *cm*^−2^]	0.4–4.5	LHS
*AOT*	Aerosol Optical Thickness	unitless	0.05–0.5	LHS
*ALPHA*	Angstrom coefficient	unitless	0.05–2	LHS
*G*	Henyey-Greenstein asymmetry factor	unitless	0.6–1	LHS
*Illumination*/ *observation conditions*: 4SAIL and 6SV				
*θ_s_*	Sun zenith angle	[°]	20–30	Uniform
*θ_v_*	View zenith angle	[°]	0	–
*ɸ*	Sun-sensor azimuth angle	[°]	0	–

**Table 2:** Range, mean and standard deviation (SD) for ground measurements from 2017 and 2018 MNI campaigns of leaf biochemical and structural variables: *C_ab_*, *C*_*w*_ and *C*_*m*_, as well as biophysical canopy variables: LAI, lai*C_ab_*, lai*C*_*w*_ and lai*C*_*m*_.

Variables		Units	Range	Mean	SD
Leaf variables:					
*C_ab_*	leaf chlorophyll content	[*μ*g/cm^2^]	38.5 to 60.8	52.8	6
*C_w_*	leaf water content	[cm]	0.012 to 0.025	0.019	0.003
*C_m_*	leaf dry matter content	[g/cm^2^](x1000)	2.62 to 7.58	5.03	1.38
Canopy variables:					
LAI	leaf area index	[m^2^/m^2^]	0.2 to 3.9	2.9	1.2
lai*C_ab_*	canopy chlorophyll content	[g/m^2^]	0.08 to 2.22	1.55	0.71
lai*C_w_*	canopy water content	[g/m^2^]	32.1 to 887.9	562.5	274.8
lai*C_m_*	canopy dry matter content	[g/m^2^]	5.4 to 290.7	152.9	83.5

**Table 3:** Goodness-of-fit results of estimated vs. measured crop traits at the MNI site. Results are given for the GPR model trained with the original datasets (Full) compared to the EBD optimized datasets (EBD). Variable abbreviations and units can be found in Table 2.

Variable		*C_ab_*			*C_w_*			*C_m_*			LAI			lai*C_ab_*			lai*C_w_*			lai*C_m_*	
Dataset type		Full	EBD		Full	EBD		Full	EBD		Full	EBD		Full	EBD		Full	EBD		Full	EBD
RMSE		10.07	5.87		0.0060	0.0030		0.0016	0.0009		0.44	0.37		0.33	0.14		122.51	96.09		47.99	36.51
NRMSE (%)		45.09	26.26		48.28	23.90		32.36	17.24		11.84	10.10		15.54	6.47		14.32	11.23		16.83	12.80
R^2^		0.77	0.80		0.18	0.29		0.69	0.60		0.93	0.93		0.87	0.96		0.86	0.87		0.72	0.83
