# Supplementary material for: Gaussian processes retrieval of crop traits in Google Earth Engine based on Sentinel-2 top-of-atmosphere data
Source: Remote Sens Environ. Author manuscript; Available in PMC 2022 Sep 7. (PMC7613387; doi:10.1016/j.rse.2022.112958)
Supplement: Appendix A [file EMS152681-supplement-Appendix_A.pdf]

Appendix A

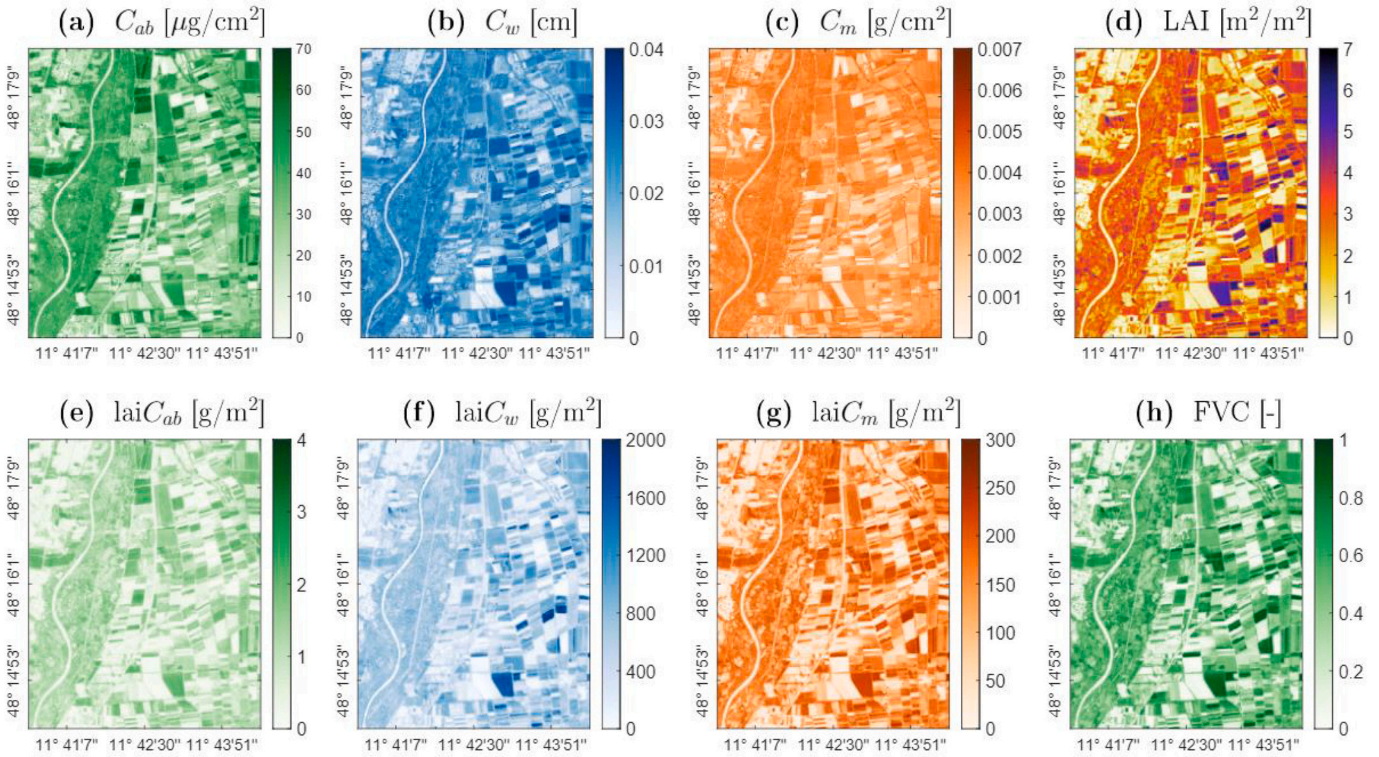

Fig. A.1. Maps (mean estimates; μ) of several crop traits: C_{ab} (a), C_w (b), C_m (c), LAI (d), laiC_{ab} (e), laiC_w (f), laiC_m (g) and FVC (h), as generated by GPR model trained over the full dataset from S2-L1C data at the MNI test site on 6 July 2017.

Table A.1
Goodness-of-fit results of estimated vs. measured crop traits at Grosseto site. Results are given for the GPR model trained with the original datasets (Full) compared to the EBD optimized datasets (EBD). Variable abbreviations and units can be found in Table 2.

Variable	C_{ab}		C_w		C_m		LAI		laiC_{ab}		laiC_w		laiC_m	
Dataset type	Full	EBD	Full	EBD	Full	EBD	Full	EBD	Full	EBD	Full	EBD	Full	EBD
RMSE	10.96	19.55	0.0104	0.0073	0.0025	0.0014	0.69	0.79	0.54	0.64	250.50	309.17	41.99	44.32
NRMSE (%)	24.85	44.33	127.97	89.96	167.94	98.25	12.39	14.21	19.22	22.72	40.61	50.12	15.75	16.63
R^2	0.01	0.07	0.23	0.35	0.37	0.36	0.78	0.78	0.58	0.65	0.74	0.75	0.74	0.78

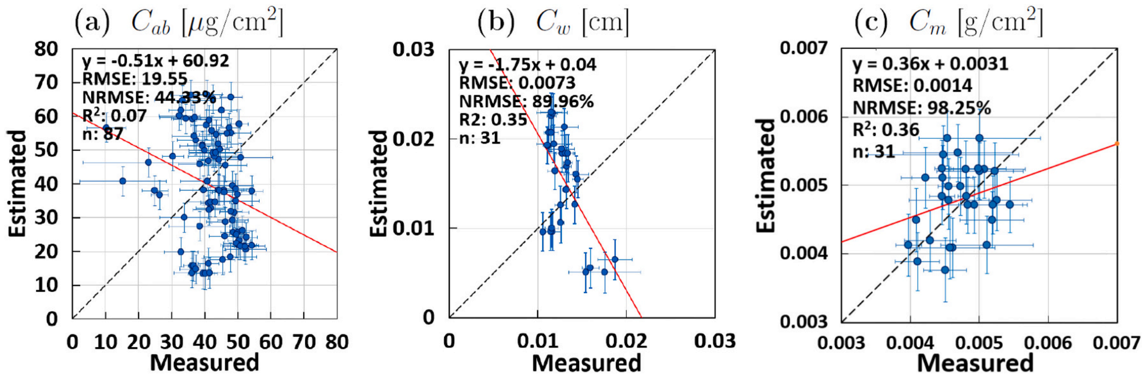

Fig. A.2. Ground validation of corn for retrieval of leaf-level crop traits over the Grosseto site by the EBD-GPR models from S2-L1C (TOA) reflectance: C_{ab} (a), C_w (b) and C_m (c). Measured vs. estimated values along the 1:1-line. Horizontal bars indicate SD for ground measurements. Vertical bars indicate associated uncertainty estimates (1 SD) for EBD-GPR model. A trend line (red) was added to represent the pattern of the points. (For interpretation of the references to color in this figure legend, the reader is referred to the web version of this article.)
